# Prevalence of depression and anxiety with premature ejaculation and its four subtypes: a systematic review and meta-analysis

**DOI:** 10.3389/fpsyt.2025.1694185

**Published:** 2026-01-05

**Authors:** Sixuan Che, Huan Sun, Yukun Kang, Xiao Hu, Fang Liu

**Affiliations:** 1 Mental Health Center, The West China Second University Hospital of Sichuan University, Chengdu, Sichuan, China; 2 Key Laboratory of Birth Defects and Related Diseases of Women and Children (Sichuan University), Ministry of Education, Chengdu, Sichuan, China; 3 Department of Psychiatry, West China Hospital of Sichuan University, Chengdu, Sichuan, China

**Keywords:** anxiety, depression, meta-analysis, premature ejaculation, subtype analysis

## Abstract

**Background:**

Premature ejaculation (PE) is a common male sexual dysfunction and is frequently accompanied by psychological comorbidities such as anxiety and depression. However, the extent of these mental health burdens across different PE subtypes remains unclear.

**Aims:**

The aims of this study were to estimate the pooled prevalence of anxiety and depression among men with PE and to compare prevalence patterns across the four established PE subtypes: acquired PE (APE), lifelong PE (LPE), premature‐like ejaculatory dysfunction (PLED), and natural variable PE (NVPE).

**Methods:**

We systematically searched PubMed, Embase, Web of Science, Cochrane Library, CENTRAL, and Springer databases through July 2025 for observational studies reporting anxiety and/or depression in PE populations. Pooled prevalence estimates were calculated using random‐effects meta‐analyses with Freeman–Tukey double-arcsine transformation. Heterogeneity was quantified using *I*², and subgroup and sensitivity analyses were performed. Instrument-specific subgroup and sensitivity analyses were conducted to address measurement heterogeneity. Publication bias was evaluated using funnel plots and Egger’s regression test.

**Results:**

Eighteen studies were included. The pooled prevalence was 42% (95% CI: 25%–61%) for anxiety and 41% (95% CI: 27%–56%) for depression, with substantial heterogeneity (*I*² = 99% for both). Egger’s tests did not identify small-study effects, although interpretation is limited by extreme heterogeneity. Subtype patterns were descriptive: APE showed the highest prevalence estimates (anxiety 44.7%; depression 43.0%), whereas NVPE showed the lowest (15.3% and 14.2%). Sensitivity analyses confirmed that no single study materially affected pooled results.

**Conclusion:**

Anxiety and depression are highly prevalent among men with PE, with notable variation across clinical subtypes. These findings underscore the importance of subtype-aware psychological assessment and individualized management in PE care.

**Systematic Review Registration:**

https://www.crd.york.ac.uk/prospero/, identifier CRD42024538434.

## Introduction

Premature ejaculation (PE) is one of the most common male sexual dysfunctions. Complaint-based population surveys typically report lifetime prevalence rates above 20% among adult men, whereas the International Society for Sexual Medicine (ISSM)-defined point prevalence is substantially lower and varies across age groups and populations (1–3). Men with PE are more likely associated with psychological disturbances such as depression, anxiety, and other measures of psychological distress than men without PE (2, 4–6). Despite extensive research, controversies persist regarding the definition, classification, and diagnostic thresholds of PE (7–9). ISSM defines lifelong and acquired PE based on three key clinical components: short intravaginal ejaculatory latency time (IELT), difficulty in delaying ejaculation, and negative personal consequences such as distress or frustration (7, 10, 11). Building on this foundation, the 2025 European Association of Urology (EAU) guideline further emphasizes the integration of both objective (e.g., IELT) and subjective indicators (e.g., perceived control and distress), providing an updated consensus beyond earlier complaint-based models (12). To address this disparity, Waldinger and Schweitzer introduced a novel classification system for PE, delineating four distinct subtypes, namely, acquired PE (APE), lifelong PE (LPE), premature-like ejaculatory dysfunction (PLED; also termed subjective PE), and natural variable PE (NVPE) (4, 13, 14). Recent consensus statements and clinical evidence have refined these criteria, highlighting IELT cutoffs around 1 min for LPE and approximately 3 min for APE, while emphasizing the subjective dimensions of control and distress (12, 15). Moreover, contemporary work has linked impaired impulse control with shorter ejaculation time and greater psychiatric symptom burden in men with PE, suggesting overlapping neurobehavioral mechanisms (16).

Despite the extensive body of research investigating the relationship between depression and anxiety with PE, findings across various studies have yielded inconsistent results. Althof et al. (7) observed that patients with APE exhibited elevated levels of anxiety and depression, but there is no consensus about the psychological disturbances with different types of PE. Even though it is commonly acknowledged that PE has a complex etiology that includes urologic, neurological, endocrine, mental, hereditary, and other variables, and both pharmacological and psychotherapy interventions are appropriate (17), different types of PE should be treated with different emphasis.

The primary objectives of this systematic review and meta-analysis are to quantitatively evaluate all eligible observational studies that investigate the association between PE and depression and anxiety, and focus on the relationship between these psychological disorders and four different types of PE additionally, thereby filling the research gap, providing a more comprehensive and accurate understanding of this association, and improving the outcomes of treatment of patients with PE.

## Methods

### Search strategy and study identification

This systematic review and meta-analysis was conducted in accordance with the PRISMA 2020 guidelines. A comprehensive literature search was conducted across the following databases from inception to 1 July 2025: PubMed, Embase, Web of Science, Cochrane Library, CENTRAL, Springer, and Grey literature sources (e.g., OpenGrey). The search strategy included MeSH terms and text words related to “premature ejaculation”, “subtypes”, “depression”, “anxiety”, and “prevalence”. Boolean operators and wildcard truncations (e.g., *depress*, *anxiety*, and *ejaculate*) were applied to ensure sensitivity. The full search strategies are provided in **Supplementary Table S1**.

Only English-language articles were included. Although language restriction may introduce bias, resource constraints and the lack of standardized translations justified this decision, which is acknowledged in the limitations section.

Additionally, we manually screened reference lists of relevant reviews and included articles to identify additional eligible studies. This study was prospectively registered in PROSPERO (CRD42024538434).

### Eligibility criteria

Studies were included if the following criteria were met: (a) Population: patients >18 years of age diagnosed with PE, with PE subtypes clearly classified as APE, LPE, PLED, or NVPE, based on Waldinger or ISSM criteria; (b) Study design: observational studies, including cross-sectional and cohort designs; and (c) Outcomes: studies reporting the prevalence of anxiety and/or depression, measured using validated instruments (e.g., GAD-7, PHQ-9, HADS, BDI, and SDS), and providing sufficient data to calculate pooled proportions.

In contrast, exclusion criteria were applied to the following: (a) non-original research (reviews, editorials, commentaries, and conference abstracts); (b) studies without validated screening instruments for depression or anxiety; (c) studies lacking extractable data or full-text availability; (d) non-English publications; and (e) duplicate reports or studies based on overlapping populations (in such cases, the most recent or comprehensive report was retained).

### Study selection and data extraction

Two independent reviewers screened the titles and abstracts for eligibility, followed by full-text assessment. Discrepancies were resolved by discussion or a third reviewer. For included studies, the following data were extracted using a standardized form: author, year, country, study design, sample size, participant age, diagnostic criteria for PE, mental health assessment tools, PE subtype classification, and outcome data.

### Risk of bias assessment

Given that all included studies were observational in nature, we assessed methodological quality using an adapted version of the Newcastle–Ottawa Scale (NOS) that has been applied in previous meta-analyses of observational prevalence studies (18–20). This adaptation retains the core NOS domains but condenses them into five equally weighted items: (1) sample representativeness, (2) sample size adequacy, (3) comparability between respondents and non-respondents, (4) validity of depression/anxiety assessment, and (5) statistical reporting quality. Each item was scored 1 point if the criterion was fully met and 0 otherwise, yielding a total score ranging from 0 to 5. The maximum score was 5 points, with studies scoring ≥3 considered to be at low risk of bias, and <3 considered high risk.

Two reviewers independently assessed the risk of bias. Disagreements were resolved through discussion or adjudication by a third reviewer. Detailed results of quality assessment are available in **Table 1**.

**Table 1:** Characteristics of 18 studies included in the meta-analysis.

Study	Study design	Country	Sample size	Age (years), mean ± SD (range)	Diagnosis of PE	Assessment of anxiety/depression	NOS
Mourikis et al., 2015	Cross-sectional	Greece	26	37.9 ± 10.1	DSM-IV-TR	STAI/BDI	3
Corretti et al., 2006	Cross-sectional	Italy	52	43.25 ± 13.11	DSM-IV-TR	SCID	1
Yang et al., 2019	Cross-sectional	China	541	37.93 ± 4.68	Unsatisfying ejaculation time, PEDT	GAD-7/PHQ-9	5
Zhang et al., 2013	Cross-sectional	China	1988	35.52 ± 10.38	Unsatisfying ejaculation time, Waldinger and Schweitzer classification	SAS/SDS	4
Demirci et al., 2023	Cross-sectional	Turkey	112	50.59 ± 13.42	ISSM definition, PEDT	HADS	2
Culha et al., 2020	Cohort	Turkey	53	42.41 ± 11.14 (22–60)	ISSM definition, PEP	STAI/BDI	2
Chierig et al., 2020	Cross-sectional	Italy	133	47.0 (36, 56)	PEDT ≥ 11	BDI	4
Zhang et al., 2022	Cross-sectional	China	203	31.00 ± 4.32	IELT ≤ 3 min	GAD-7/PHQ-9	5
Gao et al., 2013	Cross-sectional	China	778	37.15 ± 10.42	Waldinger and Schweitzer classification	SAS/SDS	4
Zhang et al., 2013	Cross-sectional	China	1206	≥18 years	ISSM definition	SDS	5
Cao et al., 2019	Cross-sectional	China	86	≥18 years	PEDT ≥ 11	HADS	4
McCabe et al., 2014	Cross-sectional	Australia	180	18–65	PEP, DSM-IV-TR, IELT ≤1 min	DASS-21	2
Kalra et al., 2015	Cross-sectional	India	55	18–50	DSM‐IV TR, ASEX	DSM-IV-TR	1
Vivekanandan et al., 2019	Cross-sectional	India	41	45.07 ± 13.46	CIPE‐5	CIS-R	2
Mialon et al., 2012	Cross-sectional	Switzerland	283	19.53 ± 1.27	The PEPA survey	MDI	4
Rajkumar et al., 2014	Cross-sectional	India	28	31.86 ± 5.92	DSM-IV, Waldinger and Schweitzer classification	ICD-10	1
Lu et al., 2020	Cross-sectional	China	688	50–70	PEDT	GAD-7/PHQ-9	4
Porst et al., 2007	Cross-sectional	United States, Germany, Italy	2,754	42.32 ± 13.13	The PEPA survey	The PEPA survey	3

PEDT, Premature Ejaculation Diagnostic Tool; ISSM, International Society for Sexual Medicine; PEP, Premature Ejaculation Profile; IELT, Intravaginal Ejaculatory Latency Time; ASEX, Arizona Sexual Experiences Scale; CIPE-5, Chinese Index of Premature Ejaculation; PEPA: Premature Ejaculation Prevalence and Attitude; NOS: Newcastle–Ottawa score; STAI, State-Trait Anxiety Inventory; BDI, Beck Depression Inventory; SCID, Structured Clinical Interview for DSM-IV Axis I Disorders; GAD‐7, General Anxiety Disorder‐7; PHQ‐9, Patient Health Questionnaire‐9; SAS, Zung self-rating anxiety scales; SDS, Zung self-rating depression scales; HADS, Hospital Anxiety and Depression Scale; DASS-21, 21-item version of the Depression Anxiety Stress Scales; CIS-R, Revised Clinical Interview Schedule; MDI, Major Depression Inventory.

### Statistical analysis

All statistical analyses were performed using R software (version 4.3.0) and RevMan 5.4.1. For each outcome (anxiety and depression), pooled prevalence estimates were calculated using a random-effects model to account for anticipated heterogeneity across studies due to variation in study populations, PE subtype definitions, and psychological assessment tools (21). Because prevalence data may exhibit variance instability—particularly when proportions approach 0 or 1—pooled prevalence was estimated using the metaprop function in the metafor package in R, applying the Freeman–Tukey double-arcsine transformation to stabilize variances across the full 0%–100% range. Standard normal (*z*)-based inference was used, as the number of studies included in each analysis exceeded common thresholds for *z*-approximation. Zero-event studies were retained without continuity corrections because the Freeman–Tukey method accommodates proportions of 0% or 100% and provides stable variance estimates. Between-study variance (τ²) was estimated using a random-effects model with REML, consistent with the high heterogeneity observed (*I*² ≥ 75%). After pooling, transformed estimates were back-transformed to the original proportion scale using the inverse Freeman–Tukey method.

Heterogeneity was quantified using Cochran’s *Q* test and the *I*² statistic, with *I*² values of 25%, 50%, and 75% considered low, moderate, and high heterogeneity, respectively. An *I*² value ≥75% was considered to indicate substantial heterogeneity (22).

To further explore potential contributors to heterogeneity, we performed influence diagnostics (leave-one-out refitting and influence plots) and exploratory random-effects meta-regression using REML estimation. Although multiple prespecified moderators were considered—including mean age, IELT thresholds, diagnostic criteria (ISSM *vs*. complaint-based), psychological instrument type/cutoff, geographic region, and NOS score—only mean age could be included, as it was the only variable consistently reported across all studies. Other moderators exhibited substantial missingness or inconsistent definitions that prevented model convergence. Consequently, meta-regression was restricted to a univariable model with mean age as the moderator.

Subgroup analyses were further performed according to PE subtype (APE, LPE, PLED, and NVPE). Because included studies reported dichotomized subtype-specific prevalence, odds ratios (ORs) were used as the contrast measure to enable consistent between-subtype comparisons. Alternative approaches for proportion differences (e.g., logit transformations, double-arcsine methods, and GLMMs) required complete binomial data for each subtype, which were not uniformly available. Between-group differences were assessed using the *Q*-between statistic.

Given the use of multiple validated psychological instruments with non-equivalent cutoff thresholds (e.g., HADS-A, GAD-7, SAS, and STAI), we additionally conducted instrument-specific subgroup meta-analyses to address measurement heterogeneity. For each instrument category, pooled prevalence was estimated using the same Freeman–Tukey transformation and REML random-effects model as in the primary analysis. Within each measurement subgroup, leave-one-out sensitivity analyses were performed to assess the influence of individual studies.

In addition, the impact of study quality on pooled estimates was examined using NOS-based sensitivity analyses. All studies were stratified into low-risk (NOS ≥ 3) and high-risk (NOS < 3) tiers, and random-effects meta-analyses were conducted within each tier to compare the distribution of effect sizes across quality levels. We further repeated the meta-analysis after excluding all high-risk studies to evaluate whether restricting the dataset to low-risk studies materially changed the overall pooled proportion.

Global leave-one-out influence analyses were additionally performed, in which each study was removed sequentially to examine its effect on the overall pooled estimates. The robustness of findings was assessed by evaluating the stability of pooled values and the overlap of corresponding confidence intervals.

Publication bias was assessed via visual inspection of funnel plots and Egger’s regression test. A two-tailed *p*-value < 0.05 was considered statistically significant.

## Results

### Study characteristics and quality assessment

**Figure 1:**
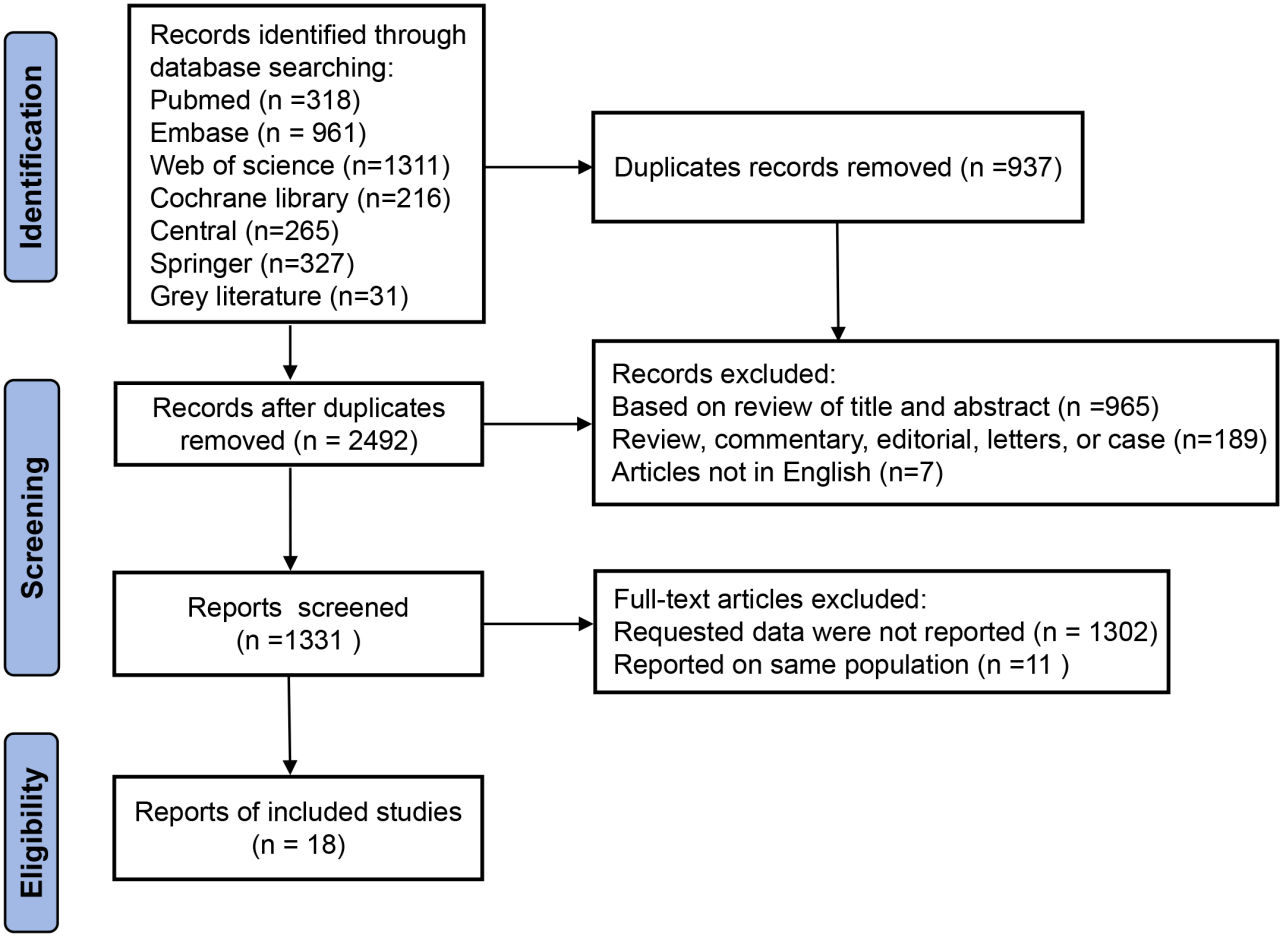
PRISMA flow diagram outlining the study selection process.

The initial literature search across multiple English-language databases yielded a total of 3,429 records. After removing 937 duplicates using reference management software, 2,492 unique articles remained. During title and abstract screening, 1,161 studies were excluded for being clearly irrelevant to the research topic. The remaining 1,331 articles were subjected to full-text screening, of which 1,313 studies were excluded due to reasons such as lack of relevant outcome data, inappropriate study design, or use of non-standardized assessment tools. Ultimately, 18 studies met the inclusion criteria and were included in the meta-analysis. The detailed study selection process is illustrated in **Figure 1**, and the characteristics of the included studies are summarized in **Table 1**.

The methodological quality of the 18 included studies was assessed using a modified version of NOS (**Table 1**). Scores ranged from 1 to 5, with 11 studies scoring ≥3 points, indicating a low risk of bias, and studies scoring 1–2 points suggest moderate to high risk of bias.

High-quality studies, such as those by Lu et al. (2020), Yang et al. (2019), and Zhang et al. (2022), demonstrated good representativeness and use of validated psychiatric instruments. Lower-quality studies, including Corretti et al. (2006) and Kalra et al. (2015), were limited by small sample sizes or unclear assessment criteria. Although all included studies were observational in design (17 cross-sectional and 1 cohort), most reported standardized methodologies and outcomes. Overall, the included studies were deemed to have acceptable methodological quality to support further meta-analysis.

### Prevalence of depression and anxiety in PE

**Figure 2:**
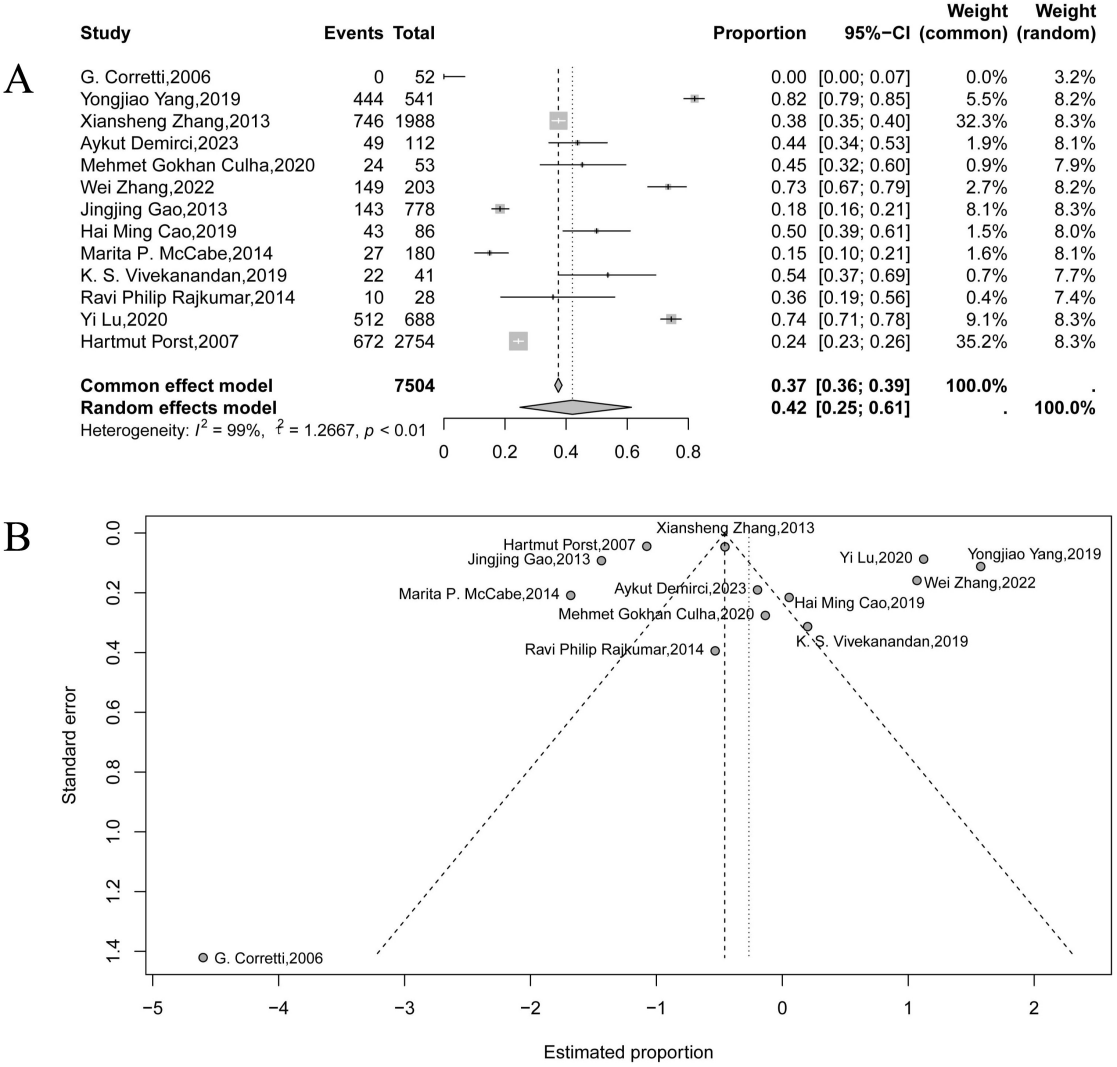
Forest plot **(A)** and funnel plot **(B)** showing the pooled prevalence of anxiety among patients with premature ejaculation.

Based on the pooled data from 13 studies, the overall prevalence of anxiety in patients with PE was estimated at 42% (95% CI: 25%–61%), as shown in **Figure 2A**. However, substantial between-study heterogeneity was observed (τ² = 1.27, *I*² = 99%, *p* < 0.01), consistent with wide variability across study populations and assessment tools. The funnel plot (**Figure 2B**) showed visible asymmetry; however, given the extremely high heterogeneity and the known limitations of funnel plots for proportion data, this pattern should be interpreted with caution and does not necessarily indicate publication bias.

**Figure 3:**
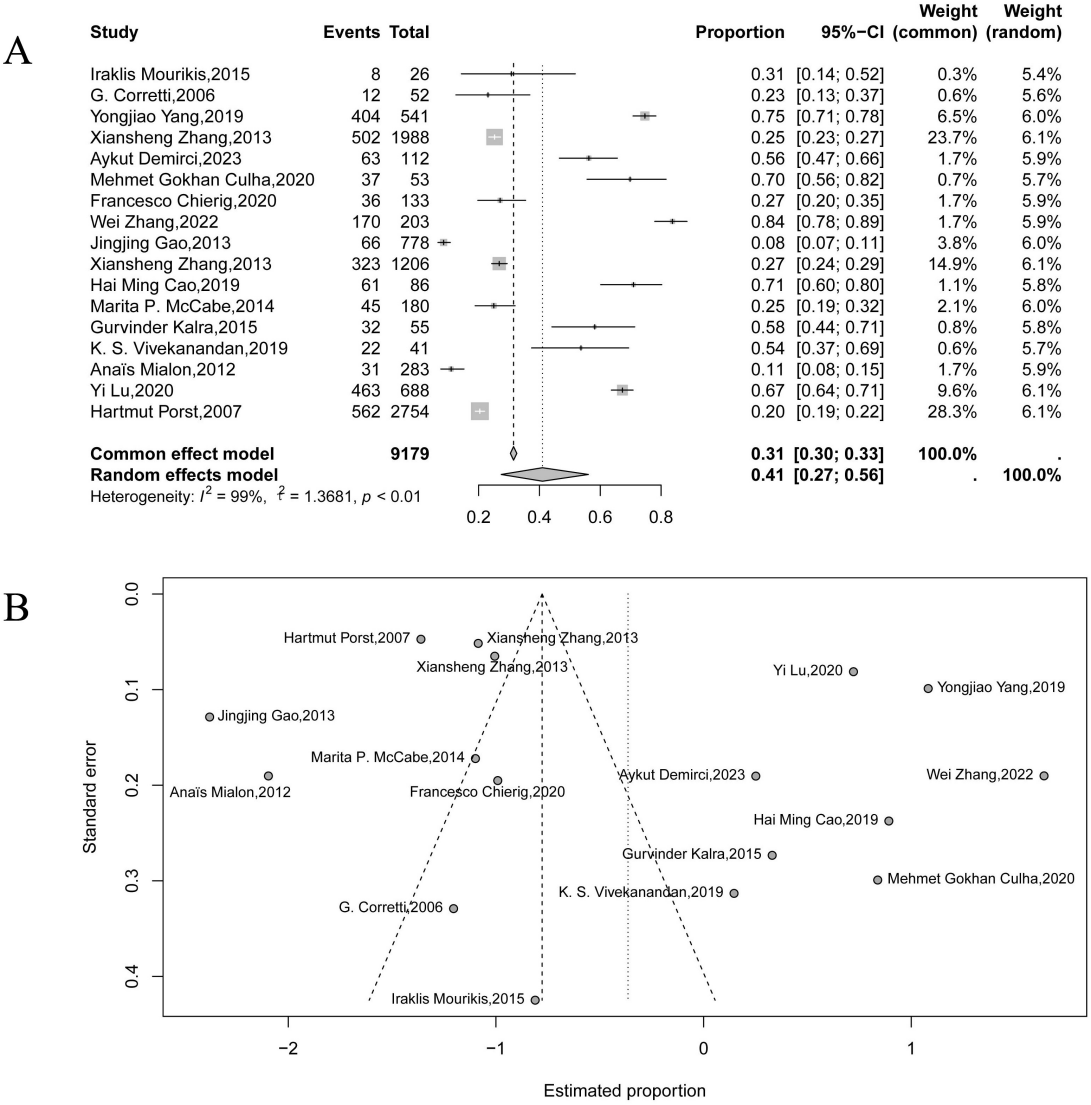
Forest plot **(A)** and funnel plot **(B)** showing the pooled prevalence of depression among patients with premature ejaculation.

Similarly, the pooled prevalence of depression was found to be 41% (95% CI: 27%–56%), according to the 16 included studies (**Figure 3A**). Substantial heterogeneity was again present (τ² = 1.37, *I*² = 99%, *p* < 0.01). The funnel plot (**Figure 3B**) showed notable asymmetry; however, given the extremely high heterogeneity and the known limitations of funnel plots for proportion data, this pattern should be interpreted cautiously and does not necessarily indicate publication bias.

The marked heterogeneity in both analyses may stem from differences in participant demographics (e.g., age, culture, and geographic location), inconsistent diagnostic criteria for PE and psychological comorbidities, and the variability in study design and assessment tools. Additionally, differences across PE subtypes (e.g., APE, LPE, NVPE, and PLED) may contribute to these variations in prevalence estimates. Given the substantial heterogeneity observed in the primary analyses, we further examined whether variability in psychological assessment tools contributed to these differences. For anxiety, instrument-specific subgroup meta-analyses using GAD-7, HADS-A, SAS, and STAI produced distinct pooled estimates (**Supplementary Figure S1**). GAD-7 showed a relatively high prevalence of 0.77 (95% CI: 0.63–0.87; *I*^2^ = 83%), HADS-A produced a moderate estimate of 0.46 (95% CI: 0.15–0.81; *I*² = 0%), SAS yielded a lower estimate of 0.27 (95% CI: 0.00–0.99; *I*² = 99%), and STAI, based on a single study, produced an estimate of 0.45 (95% CI: 0.32–0.60). These differences indicate that measurement instruments and cutoff thresholds contributed to heterogeneity in anxiety estimates. Instrument-specific leave-one-out analyses (**Supplementary Figure S2**) further confirmed that no single study exerted undue influence on subgroup results. A similar pattern was observed for depression (**Supplementary Figures S3**, **S4**). Subgroup meta-analyses using BDI/Beck, HADS-D, PHQ-9, and SDS showed substantial variability under the random-effects model, with estimates ranging from 0.18 (95% CI: 0.03–0.60; *I*^2^ = 98%; SDS) to 0.75 (95% CI: 0.50–0.91; *I*^2^ = 91%; PHQ-9). Estimates for HADS-D and BDI/Beck were 0.64 (95% CI: 0.03–0.99; *I*² = 77%) and 0.42 (95% CI: 0.06–0.90; *I*^2^ = 93%), respectively. Instrument-specific sensitivity analyses showed stable pooled estimates across all subgroups, indicating that measurement variability contributed to overall heterogeneity without altering the direction of the main findings.

For both anxiety and depression, the forest plots (**Supplementary Figure S5**) showed wide variation in effect sizes and consistently high heterogeneity, with studies ranging from strongly positive to null or negative estimates. To explore this heterogeneity, we conducted random-effects meta-regression using mean age as the only available moderator. In both anxiety and depression analyses (**Supplementary Figure S6**), regression lines were nearly flat with confidence intervals crossing zero, indicating no moderating effect of age. Overall, the meta-regression confirmed that mean age did not account for the substantial between-study heterogeneity, which likely reflects unmeasured differences in study populations, settings, or psychological assessment methods.

### Subgroup analysis by PE subtypes

To explore sources of heterogeneity, we conducted subgroup analyses stratified by the four validated subtypes proposed by Waldinger and Schweitzer—LPE, APE, NVPE [also termed variable PE (VPE)], and PLED. Studies originally reporting “PE” without subtype specification were excluded from this analysis or reclassified based on diagnostic descriptions (e.g., IELT < 1 min for LPE; onset after normal function for APE; fluctuating IELT ≥ 3 min for NVPE; subjective rapid ejaculation without IELT abnormality for PLED) following ISSM and Waldinger criteria.

**Figure 4:**
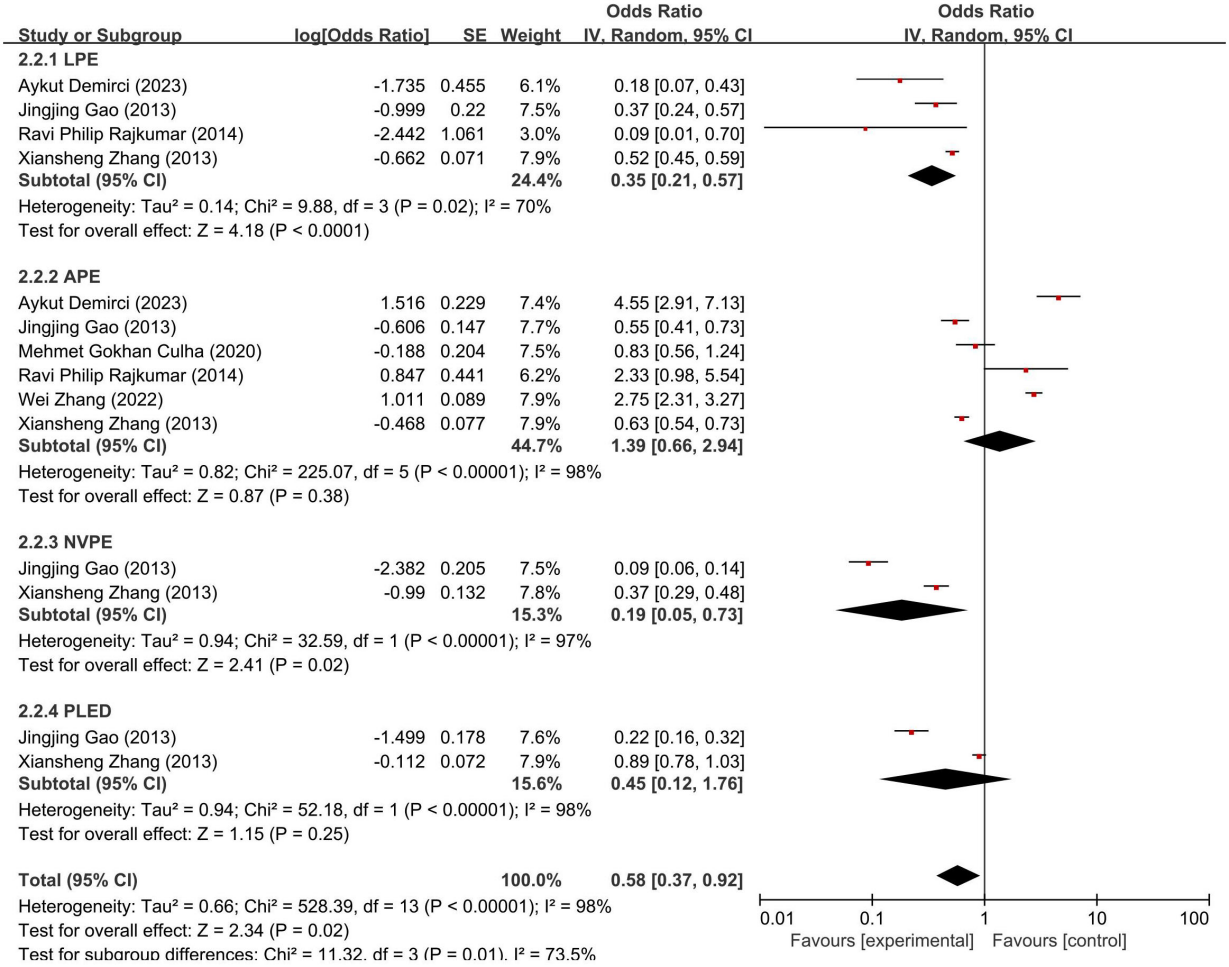
Forest plot of subgroup meta-analysis showing odds ratios (ORs) and 95% confidence intervals (CIs) for anxiety prevalence across four PE subtypes: LPE, APE, NVPE, and PLED.

As shown in **Figure 4**, the pooled ORs for anxiety varied numerically across subtypes—LPE (OR = 0.35, 95% CI: 0.21–0.57), APE (OR = 1.39, 95% CI: 0.66–2.94), NVPE (OR = 0.19, 95% CI: 0.05–0.73), and PLED (OR = 0.45, 95% CI: 0.12–1.76). Although APE showed the highest numerical estimate, the wide confidence interval crossing 1.0 indicates that this difference is not statistically significant. The omnibus *Q*-between statistic (χ² = 11.32, df = 3, *p* = 0.01; *I*² = 73.5%) suggests heterogeneity across subgroups; however, no inferential conclusions can be drawn regarding specific subtype contrasts because *post-hoc* pairwise comparisons were not performed. Descriptive information on *k*, sample sizes, event counts, and τ²/*I*² values is provided in **Figure 4**.

**Figure 5:**
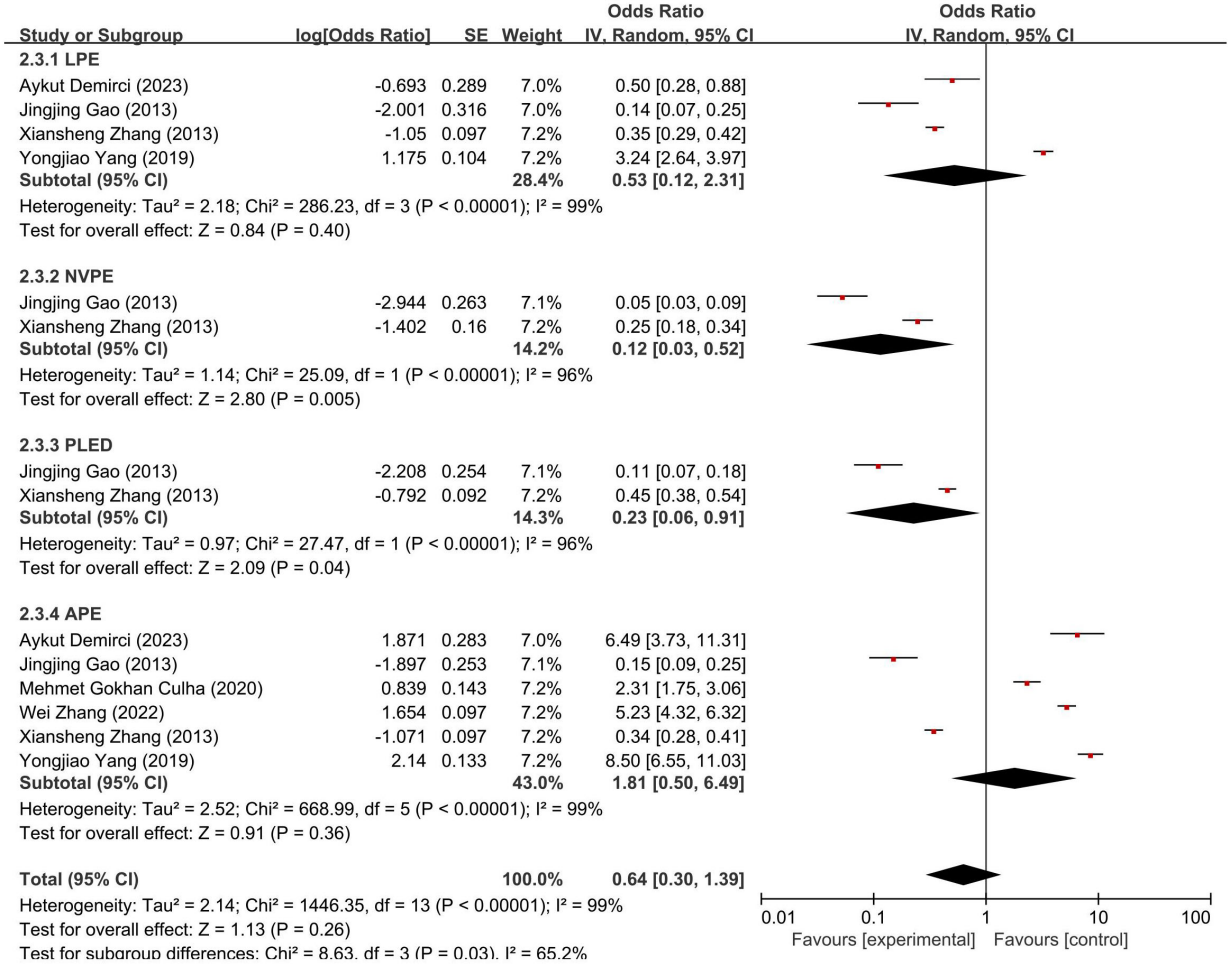
Forest plot of subgroup meta-analysis showing ORs and 95% CIs for depression prevalence across PE subtypes.

Similarly, **Figure 5** shows numerical variation in depression across subtypes—LPE (OR = 0.53, 95% CI: 0.12–2.31), APE (OR = 1.81, 95% CI: 0.50–6.49), NVPE (OR = 0.12, 95% CI: 0.03–0.52), and PLED (OR = 0.23, 95% CI: 0.06–0.91). Although the omnibus test reached significance (χ² = 8.63, df = 3, *p* = 0.03; *I*² = 65.2%), specific between-subtype differences cannot be inferred without formal *post-hoc* testing. Therefore, these results should be interpreted as descriptive patterns rather than definitive comparative evidence.

These patterns suggest that PE subtypes may exhibit differential psychological profiles, particularly in anxiety comorbidity. They further underscore the importance of subtype-specific approaches in clinical assessment and intervention planning.

### Sensitivity analysis

**Figure 6:**
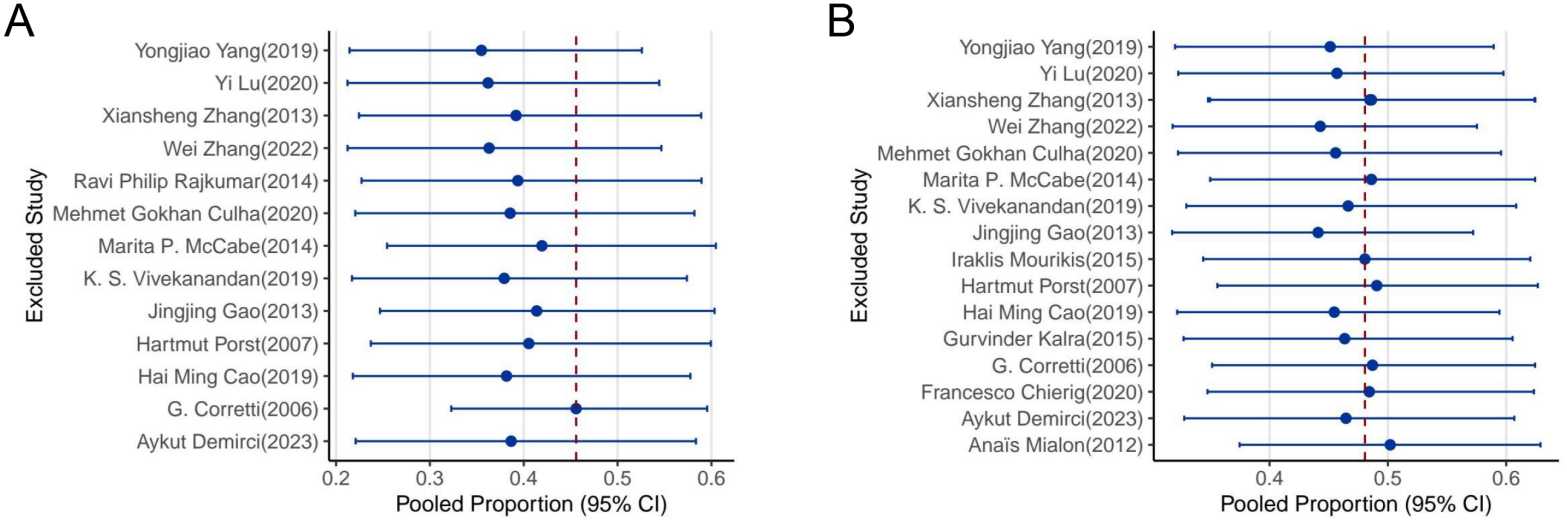
Sensitivity analysis using the leave-one-out approach. **(A)** Pooled prevalence of anxiety after excluding each individual study. **(B)** Pooled prevalence of depression after excluding each individual study. Dashed red line indicates the original pooled estimate; blue dots represent recalculated estimates with corresponding 95% confidence intervals.

To assess the robustness of the pooled prevalence estimates, we conducted leave-one-out sensitivity analyses for both anxiety and depression (**Figure 6**). In this procedure, each study was sequentially excluded, and the pooled estimate was recalculated to evaluate the influence of individual studies on the overall results.

As shown in **Figure 6A**, the exclusion of any single study did not substantially alter the pooled prevalence of anxiety among patients with PE. All recalculated estimates remained within a narrow range and close to the overall pooled proportion (approximately 42.5%), indicating strong stability of the findings.

Similarly, **Figure 6B** demonstrates that the pooled estimate for depression prevalence (approximately 47.6%) was not significantly influenced by the omission of any individual study. The confidence intervals remained consistent and did not cross critical thresholds that would indicate instability or bias.

While no individual study appeared to have an undue impact on the overall results, these findings support the general robustness of the meta-analytic estimates, albeit within the context of substantial between-study heterogeneity.

Beyond the leave-one-out analyses, NOS-based quality sensitivity analyses yielded similar findings. NOS-based quality tier analyses across all 18 included studies did not reveal any systematic differences in effect-size distributions between high-risk and low-risk studies (**Supplementary Figure S7A**). When the meta-analysis was restricted to low-risk studies (NOS ≥ 3), the pooled proportion was 43% (95% CI: 38%–49%; *I*² = 89%), which was comparable in magnitude to the estimate obtained from the full dataset (**Supplementary Figure S7B**). Because these analyses pooled proportions across both anxiety and depression outcomes on a common proportional scale, this 43% estimate reflects an overall quality-stratified proportion rather than an outcome-specific prevalence.

## Discussion

Although there is a general awareness of the association between psychological distress and PE, the actual prevalence of anxiety and depression in PE remains inconsistent due to the lack of a generally accepted PE definition. According to ISSM, PE can be classified as LPE and APE. Besides these two types, NVPE and PLED were proposed by Waldinger and Schweitzer (11, 13, 14). Recent guidelines, including the 2025 EAU update, reaffirm LPE and APE as the core diagnostic categories and emphasize integrating objective latency thresholds with subjective perceptions of control and distress (9, 12). Although these subtypes differ in onset, consistency, and perceived control, their psychological profiles remain insufficiently characterized. The purpose of this systematic review and meta-analysis was to ascertain the worldwide prevalence of anxiety and depression in patients with PE and those in the subgroups.

Across the 18 included studies, the pooled prevalence of anxiety and depression among men with PE was 42% and 41%, respectively. These rates are notably higher than those reported in large-scale surveys such as the PEPA study by Porst et al., which found anxiety and depression prevalence rates of 24.4% and 20.4%, respectively (1). More recent population-based studies have similarly reported substantial psychological symptom burdens among men with PE, supporting the higher pooled prevalence observed in our analysis (2, 3). One likely explanation for this discrepancy is the variability in screening tools: self-report measures (e.g., PHQ-9 and GAD-7) typically yield higher prevalence rates than structured interviews (e.g., SCID), likely due to reporting bias or overestimation of symptoms by patients. Additionally, the definition of PE across studies varied widely, with some viewing PE as a subjective complaint rather than a clinically defined syndrome (14), further contributing to variability.

Our subgroup analyses demonstrated numerical differences in psychological comorbidity across PE subtypes. APE showed the highest pooled prevalence numerically, whereas NVPE showed the lowest, consistent with their distinct clinical profiles. However, because confidence intervals overlapped and *post-hoc* contrasts were not performed, these patterns should be interpreted descriptively rather than as statistically confirmed differences. This variation is supported by statistical analysis: for anxiety, subgroup differences were significant, suggesting that the heterogeneity partially stems from clinical differences between subtypes. In contrast, subgroup differences for depression were only marginally significant, warranting cautious interpretation. These findings imply that the psychological burden may differ systematically across PE subtypes rather than arising from random fluctuation.

The higher burden in APE may be attributed to the multifactorial etiology of this subtype, which includes physical and psychological contributors such as erectile dysfunction, prostatitis, aging, metabolic disease, and relational distress (5, 23). The interaction between APE and depression may be bidirectional—depression can impair sexual functioning, while ongoing sexual dissatisfaction may exacerbate psychological stress (24, 25). Large-scale data support this bidirectional relationship: patients with APE exhibit elevated anxiety (82.1%) and depression (74.7%) compared to controls (26) and report greater perceived stress and interpersonal conflict (27, 28). In contrast, LPE is often linked to genetic or neurobiological underpinnings rather than psychosocial triggers, which may explain its comparatively lower psychological morbidity. Notably, psychotherapy appears less effective for LPE compared to APE, for which targeted intervention addressing underlying causes (physical or emotional) may offer relief (14, 29). This has important implications for tailoring treatment strategies. Recent EAU and ISSM guidelines support subtype-tailored management, with psychological interventions emphasized for APE and pharmacological approaches preferred for LPE (30, 31). Aligning clinical practice with these principles may improve the translation of our findings into actionable patient-centered care. Beyond these psychosocial and clinical explanations, PE subtypes are also likely shaped by distinct neurobiological and hormonal mechanisms (30). Experimental and clinical studies suggest that central serotonergic pathways, particularly 5-HT1A and 5-HT2C receptor activity, have been suggested to play a role in ejaculatory control (32, 33). Inherited variation in these systems has been more strongly linked to LPE than to APE (34, 35). Endocrine factors such as testosterone, prolactin, and thyroid function may further modulate sexual arousal and mood (36, 37). These biological susceptibilities can interact with performance anxiety, relationship stress, and negative sexual experiences, creating a self-reinforcing cycle in which psychosocial stress amplifies neurobiological vulnerabilities (4, 38). This biopsychosocial framework may help explain why patients with APE exhibit higher psychological burden, whereas LPE shows a more biologically anchored pattern.

NVPE, characterized by inconsistent and situational symptoms, showed the lowest prevalence of psychological symptoms in our analysis. Waldinger previously posited that many individuals with NVPE might reflect normal variation in sexual performance or transient distress rather than a true disorder (14). Hence, these individuals may not benefit from psychotherapeutic or pharmacological treatment. In contrast, PLED involves a mismatch between subjective distress and objective latency time (IELT), often co-occurring with anxiety and depression. One study found that patients with PLED reported higher rates of psychological distress than patients with APE (27).

Despite the robust overall estimates, the substantial heterogeneity observed in both anxiety and depression analyses warrants attention. Sensitivity analyses confirmed that no single study disproportionately influenced the results, supporting the stability of our pooled estimates. In addition, NOS-based quality analyses showed that both restricting the dataset to low-risk studies and stratifying by NOS score yielded estimates broadly consistent with the primary results, indicating that methodological quality alone is unlikely to explain the observed heterogeneity. However, heterogeneity likely stems from differences in sample size, study design, geographic and cultural factors, and assessment instruments. Our instrument-specific subgroup analyses supported this interpretation, as anxiety prevalence estimates varied substantially across HADS-A, GAD-7, SAS, and STAI. Despite these differences, instrument-specific sensitivity analyses showed consistent patterns within each measurement tool, indicating that measurement variability contributed to overall heterogeneity without altering the direction or interpretation of the main findings. Funnel plot asymmetry in anxiety analysis also raises the possibility of publication bias, although this was not confirmed by Egger’s test.

This review has several limitations. First, most included studies were cross-sectional, precluding any inference about the temporal or causal relationships between PE and psychological symptoms. Whether anxiety or depression predisposes individuals to PE, or whether PE secondarily contributes to psychological distress, cannot be determined from the available evidence. Prospective longitudinal studies and Mendelian randomization approaches are needed to clarify these causal directions. Second, many included studies had modest sample sizes, particularly within subtype analyses, which may limit statistical power. Third, inconsistent diagnostic criteria for PE, anxiety, and depression across studies may introduce misclassification bias and contribute to heterogeneity. Fourth, a parallel SMD meta-analysis could not be performed because most studies reported only dichotomized outcomes rather than continuous scores with mean and standard deviation. Finally, despite multiple subgroup and sensitivity analyses, residual confounding from unmeasured variables (e.g., relationship status and comorbid sexual dysfunctions) may still remain. Future research should prioritize standardized diagnostic tools, longitudinal study designs, and adequately powered multicenter cohorts to address these limitations.

## Conclusion

In summary, this is the first meta-analysis to evaluate the prevalence of anxiety and depression with PE in four types. While anxiety and depression are highly prevalent among men with PE, patients with APE appear to bear a disproportionate psychological burden. The observed heterogeneity across subtypes highlights the importance of nuanced, individualized treatment approaches rather than a one-size-fits-all model. Clinicians should consider the psychological profile of each PE subtype when designing treatment strategies, and future research should explore the effectiveness of targeted psychological and pharmacological interventions by subtype.

## Data Availability

The original contributions presented in the study are included in the article/**Supplementary Material**. Further inquiries can be directed to the corresponding author.
