# Supplementary material for: Prevalence of depression and anxiety with premature ejaculation and its four subtypes: a systematic review and meta-analysis
Source: Front Psychiatry. 2026 Jan 5;16:1694185. doi: 10.3389/fpsyt.2025.1694185 (PMC12812590; doi:10.3389/fpsyt.2025.1694185)
Supplement: Supplementary file 1 [file DataSheet1.docx]

# Supplementary Table S1. Study-level Data Extraction Table

| Study | Country | Setting | Sample Size | Age | PE Definition | Subtype Mapping Rule | Anxiety Instrument | Depression Instrument | NOS Domains (1-5) | NOS Score |
| --- | --- | --- | --- | --- | --- | --- | --- | --- | --- | --- |
| Mourikis et al. 2015 | Greece | Outpatient clinic | 26 | 37.9±10.1 | DSM-IV-TR | NR | STAI | BDI | 1/0/0/1/1 | 3 |
| Corretti et al. 2006 | Italy | Clinical sample | 52 | 43.25±13.11 | DSM-IV-TR | NR | SCID | SCID | 1/0/0/0/0 | 1 |
| Yang et al. 2019 | China | Hospital outpatient | 541 | 37.93±4.68 | Unsatisfying ejaculation time, PEDT | LPE/APE by PEDT + symptoms | GAD-7 | PHQ-9 | 1/1/1/1/1 | 5 |
| Zhang et al. 2013 | China | Hospital outpatient | 1988 | 35.52±10.38 | Unsatisfying ejaculation time, Waldinger classification | LPE/APE/NVPE/PLED | SAS | SDS | 1/1/1/1/0 | 4 |
| Demirci et al. 2023 | Turkey | Urology clinic | 112 | 50.59±13.42 | ISSM definition, PEDT | LPE/APE by ISSM | HADS-A | HADS-D | 1/0/1/1/0 | 2 |
| Culha et al. 2020 | Turkey | Andrology clinic | 53 | 42.41±11.14 | ISSM definition, PEP | LPE/APE by ISSM | STAI | BDI | 1/0/0/1/0 | 2 |
| Chierig et al. 2020 | Italy | Urology outpatient | 133 | 47.0 (36–56) | PEDT ≥11 | APE/LPE depending on PEDT scoring | BDI | BDI | 1/1/0/1/1 | 4 |
| Zhang et al. 2022 | China | Hospital outpatient | 203 | 31.00±4.32 | IELT≤3 min | LPE (<1), APE (<3), NVPE (>3 with fluctuation) | GAD-7 | PHQ-9 | 1/1/1/1/1 | 5 |
| Gao et al. 2013 | China | Large hospital sample | 778 | 37.15±10.42 | Waldinger classification | LPE/APE/NVPE/PLED | SAS | SDS | 1/1/1/1/0 | 4 |
| Zhang et al. 2013 | China | General hospital | 1206 | ≥18 year | ISSM definition | LPE/APE by ISSM | SDS | SDS | 1/1/1/1/1 | 5 |
| Cao et al. 2019 | China | Hospital outpatient | 86 | ≥18 year | PEDT ≥11 | APE/LPE by PEDT | HADS | HADS | 1/1/0/1/1 | 4 |
| McCabe et al. 2014 | Australia | Community sample | 180 | 18–65 | PEP, DSM-IV-TR, IELT≤1 min | LPE (<1 min) | DASS-21 Anxiety | DASS-21 Depression | 1/0/0/1/0 | 2 |
| Kalra et al. 2015 | India | Clinical sample | 55 | 18–50 | DSM-IV-TR, ASEX | NR | DSM-IV-TR | DSM-IV-TR | 1/0/0/0/0 | 1 |
| Vivekanandan et al. 2019 | India | Clinic-based | 41 | 45.07±13.46 | CIPE-5 | NR | CIS-R | CIS-R | 1/0/0/1/0 | 2 |
| Mialon et al. 2012 | Switzerland | PEPA community survey | 283 | 19.53±1.27 | The PEPA survey | Complaint-based classification | MDI | MDI | 1/1/1/1/0 | 4 |
| Rajkumar et al. 2014 | India | Clinic sample | 28 | 31.86±5.92 | DSM-IV, Waldinger classification | LPE/APE/NVPE/PLED | ICD-10 | ICD-10 | 1/0/0/0/0 | 1 |
| Lu et al. 2020 | China | Hospital outpatient | 688 | 50–70 | PEDT | APE/LPE by PEDT | GAD-7 | PHQ-9 | 1/1/1/1/0 | 4 |
| Porst et al. 2007 | USA/Germany/Italy | International survey | 2754 | 42.32±13.13 | PEPA survey | Complaint-based | PEPA survey | PEPA survey | 1/1/0/1/0 | 3 |

Supplementary Figure S1. Subgroup meta-analysis of anxiety prevalence in patients with premature ejaculation (PE) stratified by psychological assessment instrument. (A) GAD-7 subgroup: three studies reporting GAD-7–based anxiety prevalence. (B) HADS-A subgroup: two studies using the Hospital Anxiety and Depression Scale–Anxiety subscale. (C) SAS subgroup: two studies using the Self-Rating Anxiety Scale. (D) STAI subgroup: one study using the State-Trait Anxiety Inventory. For each subgroup, pooled prevalence was estimated using random-effects models. Squares represent study-level proportions, and diamond shapes represent pooled estimates. Horizontal lines indicate 95% confidence intervals.


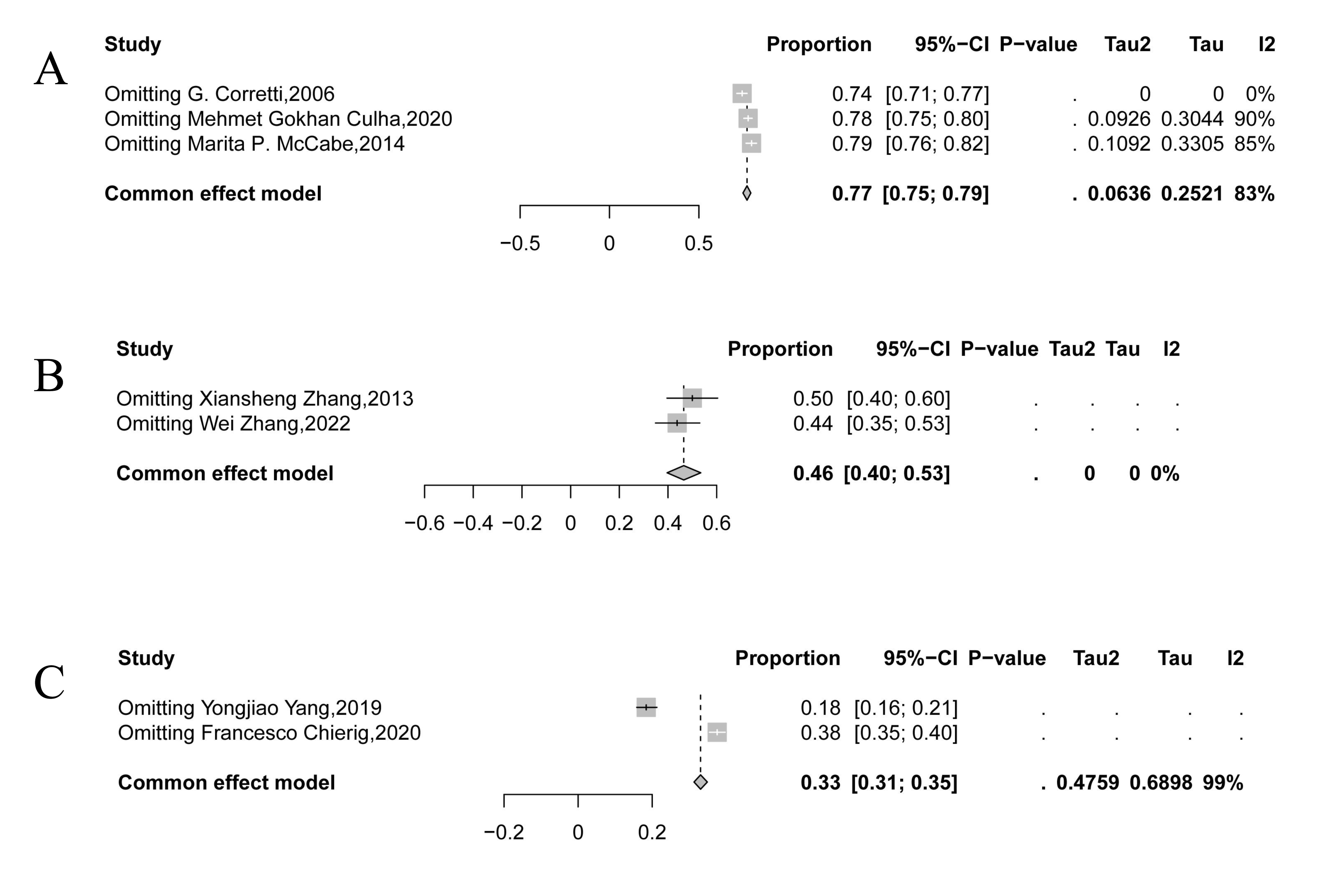


Supplementary Figure S2. Leave-one-out sensitivity analyses of anxiety prevalence stratified by psychological assessment instrument. (A) GAD-7 subgroup: sensitivity analysis excluding each study sequentially to evaluate the stability of the pooled GAD-7–based prevalence. (B) HADS-A subgroup: sensitivity analysis based on studies using the Hospital Anxiety and Depression Scale–Anxiety subscale. (C) SAS subgroup: sensitivity analysis within studies using the Self-Rating Anxiety Scale. For each subgroup, pooled estimates were re-calculated after omitting one study at a time. Diamonds represent the pooled prevalence under the common-effect model, and grey squares denote study-level estimates after exclusion.

Supplementary Figure S3. Subgroup meta-analysis of depression prevalence in patients with premature ejaculation (PE) stratified by psychological assessment instrument. (A) BDI/Beck Depression Inventory subgroup: studies using BDI-based criteria to define depressive symptoms. (B) HADS-D subgroup: studies using the Hospital Anxiety and Depression Scale–Depression subscale. (C) PHQ-9 subgroup: studies using the Patient Health Questionnaire-9. (D) SDS subgroup: studies using the Self-Rating Depression Scale. Within each subgroup, pooled prevalence estimates were calculated using random-effects models. Squares indicate study-level proportions, horizontal lines denote 95% confidence intervals, and diamonds represent pooled estimates.

Supplementary Figure S4. Leave-one-out sensitivity analyses of depression prevalence stratified by psychological assessment instrument. (A) BDI/Beck Depression Inventory subgroup: sensitivity analysis recalculating pooled prevalence after sequential omission of each included study. (B) HADS-D subgroup: sensitivity analysis for studies using the Hospital Anxiety and Depression Scale–Depression subscale. (C) PHQ-9 subgroup: sensitivity analysis for studies using the Patient Health Questionnaire-9. (D) SDS subgroup: sensitivity analysis for studies using the Self-Rating Depression Scale. Grey squares represent study-specific estimates obtained after omitting one study at a time, and diamonds indicate the corresponding pooled estimate under the common-effect model.

Supplementary Figure S5. Study-level observed effect sizes for the meta-regression analyses. (A) Anxiety group: forest plot displaying study-specific effect estimates and 95% confidence intervals used in the age-based meta-regression model. (B) Depression group: forest plot presenting study-level effect estimates and corresponding 95% confidence intervals included in the meta-regression. In both panels, squares represent individual study effect sizes, horizontal lines denote 95% confidence intervals, and the diamond indicates the pooled random-effects estimate.

Supplementary Figure S6. Bubble plots illustrating the univariable random-effects meta-regression evaluating mean age as a moderator of effect sizes. (A) Anxiety group: meta-regression of study-level effect sizes on mean age (k = 13). (B) Depression group: meta-regression of study-level effect sizes on mean age (k = 17). Each bubble represents an individual study, with bubble size proportional to study precision (1/SE). The solid line depicts the fitted regression slope, and dashed lines represent 95% confidence intervals of the regression estimate.

Supplementary Figure S7. Analyses evaluating the impact of study quality based on the Newcastle–Ottawa Scale (NOS). (A) Combined high- and low-quality studies: forest plot showing study-level log odds and corresponding 95% confidence intervals across all included studies, stratified by NOS-defined quality tiers. (B) Sensitivity analysis excluding high-risk studies: pooled prevalence estimates recalculated after omitting all studies with NOS < 3. Grey squares represent study-specific estimates obtained from sequential leave-one-out analyses, and the diamond indicates the pooled estimate under the common-effect model.
